# Bibliographical

**Published:** 1880-11

**Authors:** Joseph Richardson

**Affiliations:** Professor of the Principles of Prosthetic Dentistry in the Indiana Dental College, formerly Professor of Mechanical Dentistry and Metallurgy in the Ohio College of Dental Surgery


### BIBLIOGRAPHICAL.

### A PRACTICAL TREATISE ON MECHANICAL DENTISTRY.

*By Joseph Richardson, D.D.S., M.D., "Professor of the Principles of Prosthetic Dentistry in the Indiana Dental College, formerly Professor of Mechanical Dentistry and Metallurgy in the Ohio College of Dental Surgery." Third Edition revised and enlarged, with One Hundred and Eighty Five Illustrations.*

This work is just from the press, by Lindsay and Blakiston. It has been thoroughly revised and brought to the attainments of the present hour, in the prosthetic part of dental practice.

That which had become obsolete has been omitted, and all improved or new and valuable methods, instruments, implements, and materials, have been well and fairly presented. To those familiar with Dr. Richardson's style and method of presentation nothing need be said here upon this point except, perhaps, to affirm that this edition is better than either of the former.

In the preface to this edition the author says: "In addition to the consideration of new and improved methods and appliances of more recent introduction, as well, also, as conspicuous supplementary matters in connection with the older ones, the reader will find throughout the body of the present work not only interpolations of important facts relating to the minor details of practice, but also careful elimination of such portions of the original text as are believed, on more mature reflection, to be at variance with accepted facts and theories. This is done under the conviction that it is the province of a work like the present to preserve and perpetuate only that which survives the ordeal of experience, and to teach only that which has the sanction and approval of the enlightened judgment of the profession.

"As still occupying somewhat middle ground between condemnation and approval, the chapter on Vulcanite Base, with some modifications, has been retained in deference to many who continue to claim for it important advantages, as a cheap and convenient base. The growing distrust of its fitness, however, for this purpose, points to the conclusion that, at no distant day, it will be classified among the obsolete processes."

The preparation of this volume has involved a great amount of labor, equal in some respects to the preparation of a new work. The setting aside processes, appliances, and materials, that have been long in use and found favor in the eyes of those devoid of an investigating disposition, is not always an easy matter. And the introduction of new things, that have not been generally in use and largely tested, is not without some jeopardy to him who boldly presents and advocates them.

This may, without doubt, be regarded as the most, if not the *only*, complete text book on Prosthetic Dentistry in the English language, or any other language, so far as I am aware.

Neither the practitioner nor the student can afford to be without it. To both it will be a work for almost daily reference.

